# Fat/water separation imaging shows fatty deposition in areas of chronic left ventricular myocardial infarction

**DOI:** 10.1186/1532-429X-11-S1-O80

**Published:** 2009-01-28

**Authors:** James W Goldfarb, Margeurite Roth, Jing Han

**Affiliations:** St Francis Hospital, Roslyn, NY USA

**Keywords:** Myocardial Infarction, Full Width Half Maximum, Myocardial Segment, Prior Myocardial Infarction, Fatty Replacement

## Introduction

Several studies indicate that fatty replacement of myocardium occurs after left ventricular myocardial infarction. Magnetic resonance imaging can readily identify the location and morphology of both fatty tissue and myocardial infarction and may have the ability to non-invasively identify fatty replacement within infarcted regions. Fat deposition may impair global and regional cardiac function as well as the electrical activation of the heart.

## Methods

Twenty-five patients (patient age: 64 ± 11 yrs, infarct age: 12 ± 9 yrs) with documented prior myocardial infarctions and ten normal volunteers (age: 63 ± 10 yrs) underwent MR imaging on a clinical 1.5 T scanner using precontrast fat-water separated (Dixon) and late gadolinium-enhanced infarct imaging. Myocardial infarct location and size were assessed using the full width half maximum (FWHM) infarct sizing algorithm applied to the late gadolinium-enhanced images. Fat-water separation was performed using a three-point Dixon reconstruction from in- and opposed-phase gradient-echo images (Echo times (TEs) = 4.8, 7.2, 9.6 ms). Fat segmentation was performed on a slice by slice basis using a signal intensity threshold set two standard deviations above the noise level. Volumes of myocardial late gadolinium-enhancement and fat deposition were compared using a Student's t-test. Precontrast infarct location and detection was compared with late gadolinium-enhanced infarct imaging using a seventeen segment model.

## Results

**Figure 1:**
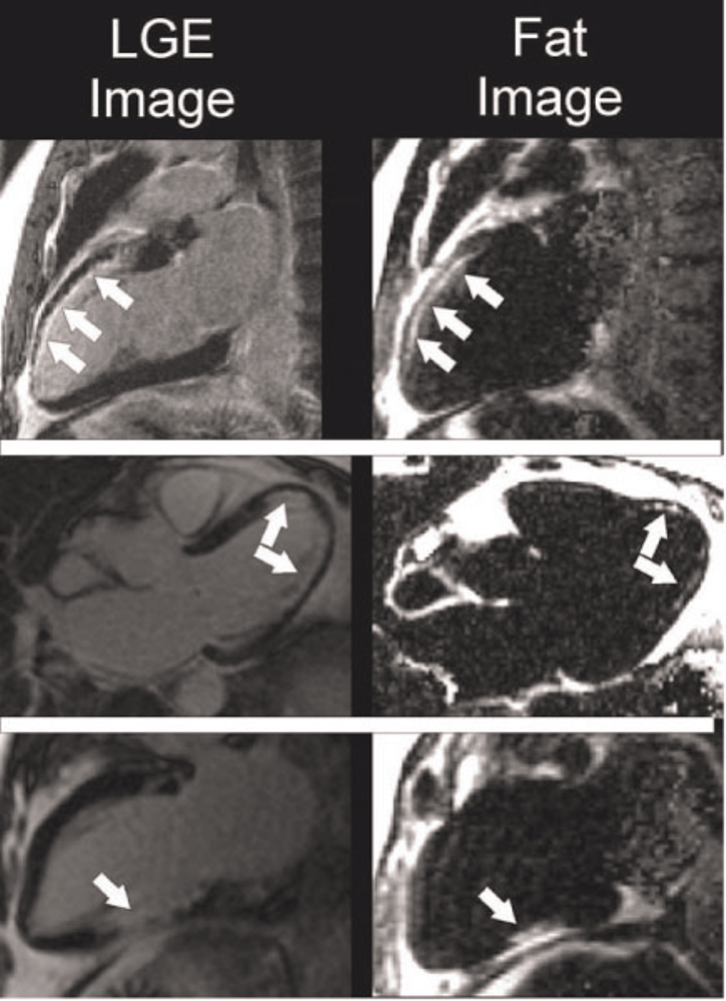
**Results from three patients show fat deposition in areas of late gadolinium-enhancement (LGE)**.

Subjects with prior myocardial infarction had a 68% prevalence of fat deposition (17/25). In subjects without a history of myocardial infarction, fat deposition and late gadolinium-enhancement were not detected (0/10). In the patients with fat deposition, the fat volume was 19.6 ± 19 ml (range: 1.7–62.6 ml) vs late gadolinium-enhancement volume of 30 ± 15 ml (range: 9.6–59.2 ml). The volumes of late gadolinium-enhancement and fat deposition were statistically different (p = 0.01). Of the 425 myocardial segments, 156 (37%) had late gadolinium-enhancement and 76 (18%) had fat deposition. 65 segments (15%) had both fat deposition and late gadolinium-enhancement, while 91 segments (21%) had late gadolinium-enhancement and no fat deposition. 11 segments (2.6%) had fat deposition and no late gadolinium-enhancement, but all 11 segments had adjacent myocardial segments with late gadolinium-enhancement. Figure 1.

## Conclusion

Fatty replacement of myocardium after myocardial infarction is common and can be readily identified using fat/water separation MR imaging. The clinical significance and biologic mechanism of fatty deposition in infarction remain to be determined.

